# Germline variants and breast cancer survival in patients with distant metastases at primary breast cancer diagnosis

**DOI:** 10.1038/s41598-021-99409-3

**Published:** 2021-10-05

**Authors:** Maria Escala-Garcia, Sander Canisius, Renske Keeman, Jonathan Beesley, Hoda Anton-Culver, Volker Arndt, Annelie Augustinsson, Heiko Becher, Matthias W. Beckmann, Sabine Behrens, Marina Bermisheva, Stig E. Bojesen, Manjeet K. Bolla, Hermann Brenner, Federico Canzian, Jose E. Castelao, Jenny Chang-Claude, Stephen J. Chanock, Fergus J. Couch, Kamila Czene, Mary B. Daly, Joe Dennis, Peter Devilee, Thilo Dörk, Alison M. Dunning, Douglas F. Easton, Arif B. Ekici, A. Heather Eliassen, Peter A. Fasching, Henrik Flyger, Manuela Gago-Dominguez, Montserrat García-Closas, José A. García-Sáenz, Jürgen Geisler, Graham G. Giles, Mervi Grip, Melanie Gündert, Eric Hahnen, Christopher A. Haiman, Niclas Håkansson, Per Hall, Ute Hamann, Jaana M. Hartikainen, Bernadette A. M. Heemskerk-Gerritsen, Antoinette Hollestelle, Reiner Hoppe, John L. Hopper, David J. Hunter, William Jacot, Anna Jakubowska, Esther M. John, Audrey Y. Jung, Rudolf Kaaks, Elza Khusnutdinova, Linetta B. Koppert, Peter Kraft, Vessela N. Kristensen, Allison W. Kurian, Diether Lambrechts, Loic Le Marchand, Annika Lindblom, Robert N. Luben, Jan Lubiński, Arto Mannermaa, Mehdi Manoochehri, Sara Margolin, Dimitrios Mavroudis, Taru A. Muranen, Heli Nevanlinna, Andrew F. Olshan, Håkan Olsson, Tjoung-Won Park-Simon, Alpa V. Patel, Paolo Peterlongo, Paul D. P. Pharoah, Kevin Punie, Paolo Radice, Gad Rennert, Hedy S. Rennert, Atocha Romero, Rebecca Roylance, Thomas Rüdiger, Matthias Ruebner, Emmanouil Saloustros, Elinor J. Sawyer, Rita K. Schmutzler, Minouk J. Schoemaker, Christopher Scott, Melissa C. Southey, Harald Surowy, Anthony J. Swerdlow, Rulla M. Tamimi, Lauren R. Teras, Emilie Thomas, Ian Tomlinson, Melissa A. Troester, Celine M. Vachon, Qin Wang, Robert Winqvist, Alicja Wolk, Argyrios Ziogas, kConFab/AOCS Investigators, Kyriaki Michailidou, Georgia Chenevix-Trench, Thomas Bachelot, Marjanka K. Schmidt

**Affiliations:** 1 Division of Molecular Pathology, The Netherlands Cancer Institute-Antoni Van Leeuwenhoek Hospital, Amsterdam, The Netherlands; 2 Division of Molecular Carcinogenesis, The Netherlands Cancer Institute-Antoni Van Leeuwenhoek Hospital, Amsterdam, The Netherlands; 3 Department of Genetics and Computational Biology, QIMR Berghofer Medical Research Institute, Brisbane, QLD Australia; 4 Department of Medicine, Genetic Epidemiology Research Institute, University of California Irvine, Irvine, CA USA; 5 Division of Clinical Epidemiology and Aging Research, German Cancer Research Center (DKFZ), Heidelberg, Germany; 6 Department of Cancer Epidemiology, Clinical Sciences, Lund University, Lund, Sweden; 7 Institute of Medical Biometry and Epidemiology, University Medical Center Hamburg-Eppendorf, Hamburg, Germany; 8 Department of Gynecology and Obstetrics, Comprehensive Cancer Center Erlangen-EMN, University Hospital Erlangen, Friedrich-Alexander University Erlangen-Nuremberg (FAU), Erlangen, Germany; 9 Division of Cancer Epidemiology, German Cancer Research Center (DKFZ), Heidelberg, Germany; 10 Institute of Biochemistry and Genetics, Ufa Federal Research Centre of the Russian Academy of Sciences, Ufa, Russia; 11 Copenhagen University Hospital, Copenhagen General Population Study, Herlev, Denmark; 12 Gentofte Hospital, Herlev, Denmark; 13 Department of Clinical Biochemistry, Copenhagen University Hospital, Herlev, Denmark; 14 Faculty of Health and Medical Sciences, University of Copenhagen, Copenhagen, Denmark; 15 Department of Public Health and Primary Care, Centre for Cancer Genetic Epidemiology, University of Cambridge, Cambridge, UK; 16 Division of Preventive Oncology, German Cancer Research Center (DKFZ) and National Center for Tumor Diseases (NCT), Heidelberg, Germany; 17 German Cancer Research Center (DKFZ), German Cancer Consortium (DKTK), Heidelberg, Germany; 18 Genomic Epidemiology Group, German Cancer Research Center (DKFZ), Heidelberg, Germany; 19 Instituto de Investigación Sanitaria Galicia Sur (IISGS), Xerencia de Xestion Integrada de Vigo-SERGAS, Oncology and Genetics Unit, Vigo, Spain; 20 University Medical Center Hamburg-Eppendorf, Cancer Epidemiology Group, University Cancer Center Hamburg (UCCH), Hamburg, Germany; 21 Division of Cancer Epidemiology and Genetics, Department of Health and Human Services, National Cancer Institute, National Institutes of Health, Bethesda, MD USA; 22 Department of Laboratory Medicine and Pathology, Mayo Clinic, Rochester, MN USA; 23 Department of Medical Epidemiology and Biostatistics, Karolinska Institutet, Stockholm, Sweden; 24 Department of Clinical Genetics, Fox Chase Cancer Center, Philadelphia, PA USA; 25 Department of Pathology, Leiden University Medical Center, Leiden, The Netherlands; 26 Department of Human Genetics, Leiden University Medical Center, Leiden, The Netherlands; 27 Gynaecology Research Unit, Hannover Medical School, Hannover, Germany; 28 Department of Oncology, Centre for Cancer Genetic Epidemiology, University of Cambridge, Cambridge, UK; 29 Institute of Human Genetics, Comprehensive Cancer Center Erlangen-EMN, University Hospital Erlangen, Friedrich-Alexander University Erlangen-Nuremberg (FAU), Erlangen, Germany; 30 Channing Division of Network Medicine, Department of Medicine, Brigham and Women’s Hospital and Harvard Medical School, Boston, MA USA; 31 Department of Epidemiology, Harvard T.H. Chan School of Public Health, Boston, MA USA; 32 Division of Hematology and Oncology, Department of Medicine, David Geffen School of Medicine, University of California at Los Angeles, Los Angeles, CA USA; 33 Department of Breast Surgery, Copenhagen University Hospital, Herlev, Denmark; 34 Instituto de Investigación Sanitaria de Santiago de Compostela (IDIS), Complejo Hospitalario Universitario de Santiago, SERGAS, Fundación Pública Galega de Medicina Xenómica, Santiago de Compostela, Spain; 35 Moores Cancer Center, University of California San Diego, La Jolla, CA USA; 36 Instituto de Investigación Sanitaria San Carlos (IdISSC), Centro Investigación Biomédica en Red de Cáncer (CIBERONC), Medical Oncology Department, Hospital Clínico San Carlos, Madrid, Spain; 37 Department of Oncology, Akershus University Hospital, Lørenskog, Norway; 38 Cancer Council Victoria, Cancer Epidemiology Division, Melbourne, VIC Australia; 39 Melbourne School of Population and Global Health, Centre for Epidemiology and Biostatistics, The University of Melbourne, Melbourne, VIC Australia; 40 Precision Medicine, School of Clinical Sciences at Monash Health, Monash University, Clayton, VIC Australia; 41 Department of Surgery, Oulu University Hospital, University of Oulu, Oulu, Finland; 42 German Cancer Research Center (DKFZ), Molecular Epidemiology Group, C080, Heidelberg, Germany; 43 Molecular Biology of Breast Cancer, University Womens Clinic Heidelberg, University of Heidelberg, Heidelberg, Germany; 44 Helmholtz Zentrum München, Institute of Diabetes Research, German Research Center for Environmental Health, Neuherberg, Germany; 45 Center for Familial Breast and Ovarian Cancer, Faculty of Medicine and University Hospital Cologne, University of Cologne, Cologne, Germany; 46 Center for Integrated Oncology (CIO), Faculty of Medicine and University Hospital Cologne, University of Cologne, Cologne, Germany; 47 Department of Preventive Medicine, Keck School of Medicine, University of Southern California, Los Angeles, CA USA; 48 Institute of Environmental Medicine, Karolinska Institutet, Stockholm, Sweden; 49 Department of Oncology, Sšdersjukhuset, Stockholm, Sweden; 50 German Cancer Research Center (DKFZ), Molecular Genetics of Breast Cancer, Heidelberg, Germany; 51 Translational Cancer Research Area, University of Eastern Finland, Kuopio, Finland; 52 Institute of Clinical Medicine, Pathology and Forensic Medicine, University of Eastern Finland, Kuopio, Finland; 53 Department of Medical Oncology, Erasmus MC Cancer Institute, Rotterdam, The Netherlands; 54 Dr. Margarete Fischer-Bosch-Institute of Clinical Pharmacology, Stuttgart, Germany; 55 University of Tübingen, Tübingen, Germany; 56 Nuffield Department of Population Health, University of Oxford, Oxford, UK; 57 Institut du Cancer de Montpellier, Montpellier University, Montpellier, France; 58 Department of Genetics and Pathology, Pomeranian Medical University, Szczecin, Poland; 59 Independent Laboratory of Molecular Biology and Genetic Diagnostics, Pomeranian Medical University, Szczecin, Poland; 60 Division of Oncology, Department of Medicine, Stanford University School of Medicine, Stanford Cancer Institute, Stanford, CA USA; 61 Department of Epidemiology & Population Health, Stanford University School of Medicine, Stanford, CA USA; 62 Department of Genetics and Fundamental Medicine, Bashkir State University, Ufa, Russia; 63 Department of Surgical Oncology, Family Cancer Clinic, Erasmus MC Cancer Institute, Rotterdam, The Netherlands; 64 Harvard T.H. Chan School of Public Health, Program in Genetic Epidemiology and Statistical Genetics, Boston, MA USA; 65 Department of Medical Genetics, Oslo University Hospital and University of Oslo, Oslo, Norway; 66 VIB Center for Cancer Biology, Leuven, Belgium; 67 Laboratory for Translational Genetics, Department of Human Genetics, University of Leuven, Leuven, Belgium; 68 University of Hawaii Cancer Center, Epidemiology Program, Honolulu, HI USA; 69 Department of Molecular Medicine and Surgery, Karolinska Institutet, Stockholm, Sweden; 70 Department of Clinical Genetics, Karolinska University Hospital, Stockholm, Sweden; 71 NIHR Biomedical Research Centre, Moorfields Eye Hospital NHS Foundation Trust and UCL Institute of Ophthalmology, London, England, UK; 72 Medical Research Council (MRC) Epidemiology Unit, University of Cambridge, Cambridge, UK; 73 Kuopio University Hospital, Biobank of Eastern Finland, Kuopio, Finland; 74 Department of Clinical Science and Education, Karolinska Institutet, Sšdersjukhuset, Stockholm, Sweden; 75 Department of Medical Oncology, University Hospital of Heraklion, Heraklion, Greece; 76 Department of Obstetrics and Gynecology, Helsinki University Hospital, University of Helsinki, Helsinki, Finland; 77 Department of Epidemiology, Gillings School of Global Public Health and UNC Lineberger Comprehensive Cancer Center, University of North Carolina at Chapel Hill, Chapel Hill, NC USA; 78 Department of Population Science, American Cancer Society, Atlanta, GA USA; 79 IFOM-The FIRC Institute of Molecular Oncology, Genome Diagnostics Program, Milan, Italy; 80 Department of General Medical Oncology and Multidisciplinary Breast Centre, Leuven Cancer Institute, University Hospitals Leuven, Leuven, Belgium; 81 Unit of Molecular Bases of Genetic Risk and Genetic Testing, Department of Research, Fondazione IRCCS Istituto Nazionale dei Tumori (INT), Milan, Italy; 82 Carmel Medical Center and Technion Faculty of Medicine, Clalit National Cancer Control Center, Haifa, Israel; 83 Medical Oncology Department, Hospital Universitario Puerta de Hierro, Madrid, Spain; 84 Department of Oncology, UCLH Foundation Trust, London, UK; 85 Institute of Pathology, Staedtisches Klinikum Karlsruhe, Karlsruhe, Germany; 86 Department of Oncology, University Hospital of Larissa, Larissa, Greece; 87 School of Cancer & Pharmaceutical Sciences, Comprehensive Cancer Centre, King’s College London, Guy’s Campus, London, UK; 88 Center for Molecular Medicine Cologne (CMMC), Faculty of Medicine and University Hospital Cologne, University of Cologne, Cologne, Germany; 89 Division of Genetics and Epidemiology, The Institute of Cancer Research, London, UK; 90 Department of Health Sciences Research, Mayo Clinic, Rochester, MN USA; 91 Department of Clinical Pathology, The University of Melbourne, Melbourne, Victoria Australia; 92 Division of Breast Cancer Research, The Institute of Cancer Research, London, UK; 93 Department of Population Health Sciences, Weill Cornell Medicine, New York, NY USA; 94 Plateforme de Bioinformatique Gilles Thomas, Centre de recherche en cancérologie de Lyon, Fondation Synergie Lyon Cancer, Université Claude Bernard Lyon 1, Lyon, France; 95 Institute of Cancer and Genomic Sciences, University of Birmingham, Birmingham, UK; 96 Wellcome Trust Centre for Human Genetics and Oxford NIHR Biomedical Research Centre, University of Oxford, Oxford, UK; 97 Division of Epidemiology, Department of Health Science Research, Mayo Clinic, Rochester, MN USA; 98 Laboratory of Cancer Genetics and Tumor Biology, Cancer and Translational Medicine Research Unit, University of Oulu, Biocenter Oulu, Oulu, Finland; 99 Laboratory of Cancer Genetics and Tumor Biology, Northern Finland Laboratory Centre Oulu, Oulu, Finland; 100 Department of Surgical Sciences, Uppsala University, Uppsala, Sweden; 101 Peter MacCallum Cancer Center, Melbourne, VIC Australia; 102 Biostatistics Unit, The Cyprus Institute of Neurology & Genetics, Nicosia, Cyprus; 103 Cyprus School of Molecular Medicine, The Cyprus Institute of Neurology & Genetics, Nicosia, Cyprus; 104 Département de Cancérologie Médicale, Centre Léon Bérard, Lyon, France; 105 Division of Psychosocial Research and Epidemiology, The Netherlands Cancer Institute-Antoni van Leeuwenhoek Hospital, Amsterdam, The Netherlands

**Keywords:** Breast cancer, Cancer genetics, Metastasis

## Abstract

Breast cancer metastasis accounts for most of the deaths from breast cancer. Identification of germline variants associated with survival in aggressive types of breast cancer may inform understanding of breast cancer progression and assist treatment. In this analysis, we studied the associations between germline variants and breast cancer survival for patients with distant metastases at primary breast cancer diagnosis. We used data from the Breast Cancer Association Consortium (BCAC) including 1062 women of European ancestry with metastatic breast cancer, 606 of whom died of breast cancer. We identified two germline variants on chromosome 1, rs138569520 and rs146023652, significantly associated with breast cancer-specific survival (P = 3.19 × 10^−8^ and 4.42 × 10^−8^). In silico analysis suggested a potential regulatory effect of the variants on the nearby target genes *SDE2* and *H3F3A*. However, the variants showed no evidence of association in a smaller replication dataset. The validation dataset was obtained from the SNPs to Risk of Metastasis (StoRM) study and included 293 patients with metastatic primary breast cancer at diagnosis. Ultimately, larger replication studies are needed to confirm the identified associations.

## Introduction

Breast cancer is the most common female cancer in the Western world and one of the most common causes of cancer death in women globally^1^. Early detection and better treatments have helped to reduce breast cancer mortality in recent decades^2^. Yet, when breast cancer metastasizes to distant sites, prognosis continues to be poor and for most cases treatment is only palliative^3^. Metastases in breast cancer can remain undetectable for many years after initial diagnosis, leading to incurable lesions^4^. Approximately 15% of patients with breast cancer will develop distant metastases within 3 years after diagnosis of the primary tumor^5^. Therefore, it is important to have the tools able to detect breast cancer metastases at earlier stages, in order to better manage and predict breast cancer progression. Prognostication models could benefit from the inclusion of germline genetic biomarkers that are capable of predicting tumor recurrence, second tumors or prognosis of second tumors. However, so far, it has been difficult to identify individual common germline variants associated with primary breast cancer survival due to the small effect size these variants are likely to have^6,7^. Likewise, evidence as to whether or not germline variants can increase the probability of metastatic progression is currently limited to a few studies^4,8^. For example, a candidate gene study identified common single nucleotide polymorphisms (SNPs) located within *SIPA1* that were associated with metastasis and poor breast cancer prognosis^9^. Other studies have identified other metastasis susceptibility genes such as *RRP1b*^10^. Germline variants could specifically provide metastatic predisposition by affecting treatment response^11^ or promoting tumor initiating events and providing new metastatic functions to tumor cells^4^.

The aim of this study was to identify associations between common germline variants and breast cancer-specific survival in patients with metastasis at primary breast cancer diagnosis. We hypothesized that germline variants might predispose to poorer survival after breast cancer metastasis, and that analyzing a set of patients with similar stage of the disease might help identify variants that do not show evidence of association in larger but more heterogeneous datasets.

## Results

We used data from the Breast Cancer Association Consortium (BCAC): the dataset comprised data from 50 studies from which follow-up information for women diagnosed with distant metastases at primary breast cancer diagnosis was available. The results were based on the meta-analysis of two genome-wide SNP arrays (iCOGS^12^ and OncoArray^13^ (see “Methods”). We analyzed variants that had a minor allele frequency (MAF) > 0.01 and an imputation quality r^2^ > 0.7 for at least one of the two arrays. Details about the individual studies, the genotyping array used and number of patients included are given in Supplementary Table 1. We analyzed the genotypes and clinico-pathological data of a total of 1062 breast cancer patients, 606 of whom died of breast cancer within 15 years of follow-up. Of these, 721 of the patients had estrogen receptor (ER)-positive disease (388 deaths) and 227 had ER-negative disease (148 deaths). All patients were women of European descent. The patients were diagnosed from 1979 to 2014 (median: 2004) and aged 26–92 (median: 60) years.

**Figure 1:**
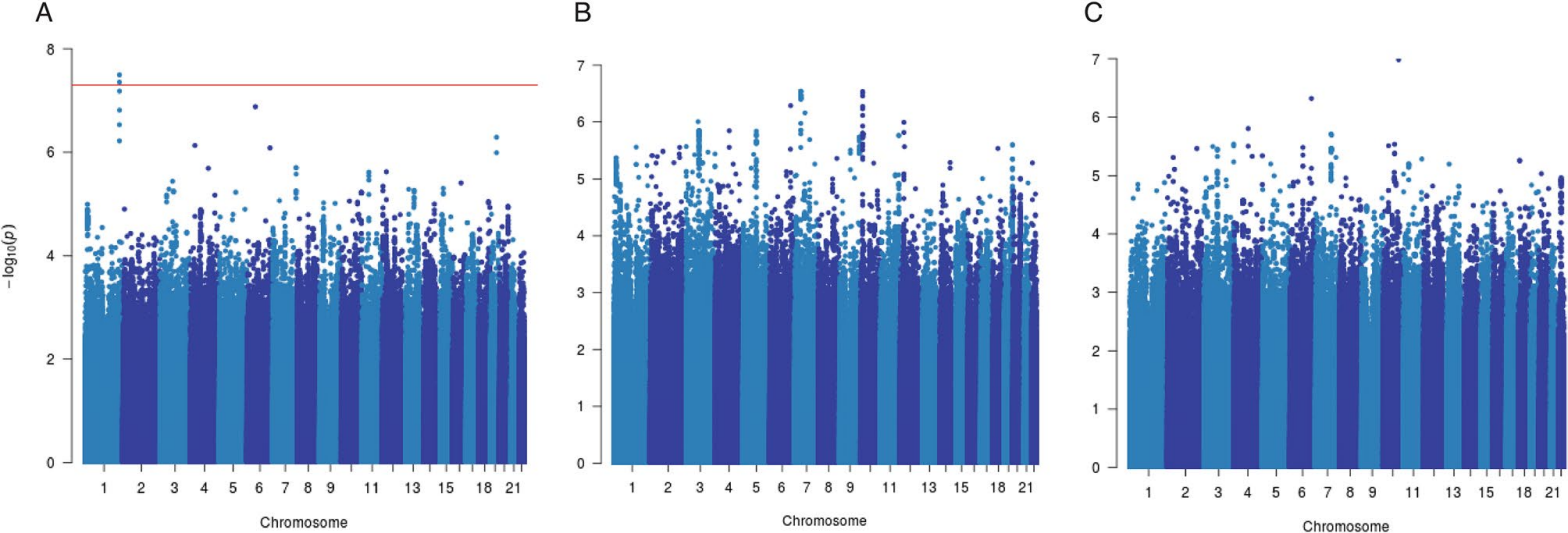
Manhattan plots of the meta-analysis of OncoArray and iCOGS datasets for the association of common germline variants and breast cancer-specific survival for patients with metastases at primary breast cancer diagnosis for (**A**) all breast tumors, (**B**) ER-positive tumors, and (**C**) ER-negative tumors. The y axis shows the − log_10_ P values of each variant analyzed, and the x axis shows their chromosome position. The red horizontal line represents P = 5 × 10^−8^.

Manhattan plots showing the association between germline variants and breast cancer-specific survival of all, ER-positive and ER-negative metastasized breast cancers are shown in Fig. 1. We identified two genome-wide significant (P < 5 × 10^−8^) variants (SNPs: rs138569520 and rs146023652) on chromosome 1 associated with breast cancer-specific survival for all metastasized breast cancers (Table 1, Supplementary Table 2). The two variants were part of a set of six highly correlated SNPs (Table 1, r^2^ > 0.88) based on European subjects in phase 3 of the 1000 Genomes Project^14^. No variant reached genome-wide significance for ER-positive or for ER-negative breast cancer tumors alone (Supplementary Tables 3 and 4).

**Table 1:** Results for the six correlated variants associated with breast cancer-specific survival for patients with metastatic primary breast cancer at diagnosis.

SNP	Chr	Position	Ref	Alt	EAF	r^2^	HR	LCL	UCL	P value
rs138569520	1	226193175	T	C	0.02	0.87	3.67	1.86	7.23	3.19 × 10^−8^
rs146023652	1	226158826	C	T	0.02	0.86	3.64	1.84	7.19	4.42 × 10^−8^
rs114512448	1	226173980	G	A	0.02	0.86	3.53	1.78	6.95	6.57 × 10^−8^
rs143653255	1	226157179	T	C	0.02	0.86	3.26	1.68	6.34	1.53 × 10^−7^
rs115086585	1	226154721	C	T	0.02	0.85	3.21	1.64	6.25	2.93 × 10^−7^
rs72757046	1	226235714	G	C	0.02	0.84	3.62	1.78	7.37	6.02 × 10^−7^

Genomic positions are based on the hg19 genome build.

*ALT* alternate, *REF* reference, *EAF* effect allele frequency, *HR* hazard rate, *LCL* lower control limit, *UCL* upper control limit, *r*^*2*^ imputation quality.

The variant with the strongest association was the SNP rs138569520 (HR = 3.67, 95% CI 1.86–7.23 and P = 3.19 × 10^−8^). The HR estimates for rs138569520 in the ER-positive (HR = 3.38, 95% CI 1.48–7.70 and P = 4.37 × 10^−4^) and ER-negative (HR = 2.76, 95% CI 1.16–6.64 and P = 8.70 × 10^−3^) were similar (P = 0.97 for difference).

**Figure 2:**
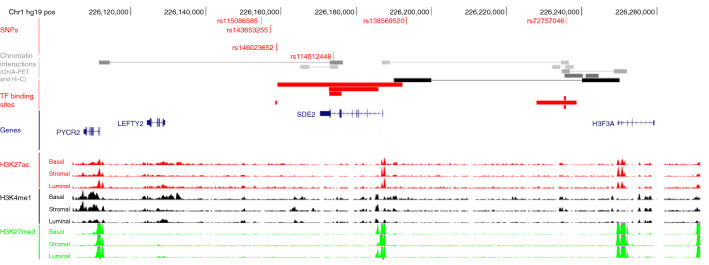
Functional annotation of the six highly correlated SNPs: rs138569520, rs146023652, rs114512448, rs143653255, rs115086585 and rs72757046. *TF* transcription factor.

Several genes (*SDE2*, *LEFTY2*, *PYCR2* and *H3F3A)* were located within 100 kb of the most significant SNP rs138569520. We interrogated functional genomic data including annotations of enhancers, promoters and transcription factor binding sites and found evidence consistent with gene regulation in the regions containing the associated variants (Fig. 2). Hi-C analysis in HMEC cells^15^ showed that the lead variant rs138569520 is located in a genomic region interacting with the promoter region of *H3F3A*. SNPs rs146023652 and rs114512448 overlapped with transcription factor (TF) binding sites which might reflect the active transcription of *SDE2.* ChIP-seq signals from primary breast sub-populations^16^ also showed potential regulatory regions containing rs114512448. ChIA-PET analysis in MCF-7 cells from ENCODE^17^, detected an interaction between rs114512448 and the *PYCR2* gene. Finally, ChIA-PET also detected an interaction between rs72757046 and *SDE2* and *H3F3A*.

**Figure 3:**
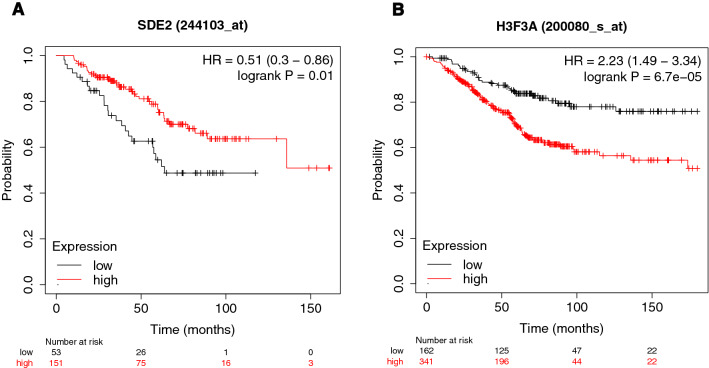
Kaplan–Meier overall survival plot for high versus low expression level of the genes (**A**) *SDE2* (n = 204) and (**B**) *H3F3A* (n = 503) restricted to patients with a grade 3 tumor and 15 years of follow-up. The differential expression analysis was performed in KMplotter.

Using KMplotter (kmplot.com/analysis)^18^, we tested the association of the mRNA tumor expression of *SDE2* and *H3F3A*, the genes in closest proximity to rs138569520, with overall survival in grade 3 breast tumors (to select the most aggressive subtype; selection for stage 4 was not available). Low mRNA expression levels of *SDE2* gene were significantly associated (P = 0.01) with poorer breast cancer survival (Fig. 3a), while, in contrast, high expression of *H3F3A* was associated with lower survival (P = 6.7 × 10^−5^) (Fig. 3b). These associations were not statistically significant, neither for grade 1 or for grade 2 disease (P > 0.21).

Lastly, we aimed to evaluate the significance of the two genome-wide significant SNPs using an independent set of 293 breast cancer patients with metastatic primary breast cancer at diagnosis from the SNPs to Risk of Metastasis (StoRM) study^19^. All patients were diagnosed in France from March 2012 to May 2014, aged 18 years or older (median: 59 years) and followed up to July 2017. A total of 293 patients were available for the validation study, 239 of whom had events, defined as progression and/or death occurring during follow-up. Both SNPs had good imputation quality (r^2^ ~ 0.7) and similar MAFs to those in the BCAC dataset (~ 2%). However, neither of the two SNPs replicated in the survival analysis with the StoRM dataset (Table 2): rs138569520 (HR = 1.49, 95% CI 0.60–3.71, P = 0.34) and rs146023652 (HR = 1.25, 95% CI 0.46–3.37, P = 0.66). Although the HR estimates in the StoRM validation dataset were smaller than those from the BCAC analyses (HR = 3.67 and 3.64), the confidence limits overlapped.

**Table 2:** Results for the validation of the two genome-wide significant variants in an independent dataset of breast cancer patients with metastatic primary breast cancer at diagnosis.

SNP	Chr	Position	Ref	Alt	EAF	r^2^	HR	LCL	UCL	P value
rs138569520	1	226193175	T	C	0.02	0.69	1.49	0.60	3.71	0.34
rs146023652	1	226158826	C	T	0.02	0.79	1.25	0.46	3.37	0.66

*ALT* alternate, *REF* reference, *EAF* effect allele frequency, *HR* hazard rate, *LCL* lower control limit, *UCL* upper control limit, *r*^*2*^ imputation quality.

Because the BCAC dataset also included prevalent cases (n = 466), we repeated the analysis with incident cases (n = 596) to match the study design in StoRM more closely. The HR estimates were similar to those for the overall analysis (rs138569520: HR = 3.77, 95% CI 1.71–8.30, P = 3.12 × 10^−5^ and rs146023652: HR = 3.75, 95% CI 1.70–8.29, P = 3.60 × 10^−5^). Finally, since the maximum follow-up in the StoRM dataset was shorter (5 years, compared with a maximum of 15 years in the BCAC dataset), we repeated the main analysis in BCAC using a follow-up of 5 years (n = 1031, 476 deaths). The associations for the two SNPs were slightly less significant (rs138569520: HR = 3.43, 95% CI 1.74–6.80, P = 1.83 × 10^−7^ and rs146023652: HR = 3.41, 95% CI 1.72–6.76, P = 2.55 × 10^−7^) but the HR estimates were similar to those from the main analysis.

## Discussion

In this analysis of breast cancer patients with metastatic primary breast cancer at diagnosis, involving 1062 patients with 606 breast cancer-specific deaths, we identified two variants on chromosome 1 (rs138569520 and rs146023652) associated with survival, at genome-wide levels of statistical significance. The most significant association was for the SNP rs138569520 (P = 3.19 × 10^−8^). The HR estimates were similar in patients with ER-positive and ER-negative disease.

Two genes, *SDE2* and *H3F3A,* were in closest proximity of rs138569520. Both genes have been previously associated with oncogenic processes relevant for metastatic progression: the *SDE2* gene (“silencing defective 2”) is known to be involved in DNA replication, telomere maintenance and cell cycle control^20,21^. The functional roles of *SDE2* have been studied in a proteome dynamics analysis in prostate cancer cells; the results suggested that alterations of the gene might diminish the error-prone DNA repair pathway activation and promote missense mutations^22^. The gene *H3F3A* encodes for histone H3.3, and mutations in this protein have been linked to multiple cancer processes^23^, including breast invasive ductal carcinoma^24^. Additionally, the differential expression of these two genes was significantly associated with survival in grade 3 tumors based on KMplotter. Previous studies have also linked the expression of these genes to oncogenic processes. For example, downregulation of *SDE2* was associated with mutation disease phenotype as well as poorer mortality outcomes^22^. Likewise, overexpression of *H3F3A* was associated with lung cancer progression and promotion of lung cancer cell migration by activation of metastasis-related genes^25^. Unfortunately, in KMplotter it was not possible to specifically select stage 4 tumors, which limits the interpretation of our findings. Future studies are needed in order to corroborate the association of *SDE2* and *H3F3A* expression with survival in this group of patients.

Additionally, there was predicted genomic activity in the locus based on the intersection of multiple genomic regulatory features in breast tissue. Although the SNPs appeared to cluster around *SDE2*, there was also *in-silico* evidence for two other potential target genes at this locus (*H3F3A* and *PYCR2*). *PYCR2* encodes for a mitochondrial protein involved in proline biosynthesis. While little is known about this proline form, studies for the close family member *PYCR1* have found that higher levels of mRNA were associated with reduced survival from breast cancer patients^26^. To support further our hypothesis that the two genome-wide significant SNPs (rs138569520 and rs146023652) were specific for survival in patients with metastatic disease, we confirmed that there were no associations (HR = 1.04, P = 0.58, MAF = 0.02 and HR = 1.03, P = 0.60, MAF = 0.02 respectively) with breast cancer-specific survival in the most recent BCAC dataset for all invasive early (stages I–III) breast cancers (OncoArray and iCOGS, n = 86,627)^27^.

On the other hand, the two genome-wide significant variants, rs138569520 and rs146023652, were not replicated (P = 0.34 and P = 0.66, respectively) using an independent dataset of patients with metastatic primary breast cancer diagnosis (n = 293). The imputation quality and the minor allele frequency of the SNPs in the replication cohort were comparable to those in the BCAC analyses (MAF = 2% and r^2^ > 7%), therefore the negative result could not be attributed to those factors. Age of the patients could also not explain the difference since both datasets had comparable median ages at diagnosis, 60 years for BCAC and 59 years for StoRM. On the other hand, it is important to state that there were several factors that varied between the datasets. First, the sample size differed considerably between BCAC (n = 1062) and the StoRM study (n = 293), the latter having a relatively small sample size which limits the power to detect associations. Total follow-up time also varied: for the BCAC dataset, patients were followed for a maximum of 15 years, while for the StoRM study the follow-up ended at 5 years. However, the results from the complementary analysis using the BCAC dataset and 5-year follow-up were comparable to the initial 15 years follow-up results. This finding suggests that the disparity in estimates between the two analyses is not due to shorter follow-up. There were several other differences between the main BCAC dataset and the StoRM cohort used for validation. For example, the BCAC dataset included multiple studies from several countries while the StoRM cohort included solely patients from France. Moreover, StoRM was a recent cohort with the earliest reported diagnosis starting in 2012. On the other hand, in BCAC, the year of patients’ diagnosis ranged between 1979 and 2014 and included prevalent cases. While the analysis in BCAC using exclusively incident cases gave comparable estimates to the main analysis, the difference in the years of diagnosis could be related to differences in treatment strategies that were not considered in the current analysis. The lack of information about detailed treatment is a potential weakness of the current analysis and validation. Treatment strategy, together with characteristics of the tumor, will also influence the final prognosis of metastatic breast cancer^28^. It is important to note that the associations observed in the BCAC study may be false positives, and that further large replication studies will be required to confirm or refute the associations.

In conclusion, this analysis of BCAC patients with metastatic primary breast cancer at diagnosis from the BCAC dataset identified a new region in chromosome 1 associated with breast cancer-specific survival. The region includes six highly correlated SNPs that are predicted to be in an active region of the genome based on in-silico evidence from breast cancer tissues and that are located in close proximity to genes involved in oncogenic processes. However, we were unable to validate the association using a smaller, independent set of patients. Overall, the role of germline variants in metastasis and progression remains unclear. Further analyses with larger datasets including treatment information and functional analysis are needed to better understand the underlying biological processes and the links between this locus and the nearby genes. Prior validation of the reported associations is needed before these findings can be used in clinical-decision making. Therefore, a next step is to study these SNPs in a, preferably, prospective large series of metastasized breast cancer patients. Ultimately, germline variants could help identifying tailored treatments for patients with metastatic disease or better strategies for risk management stratification of aggressive forms of breast cancer.

## Methods

### Breast cancer samples and genotype data: Breast Cancer Association Consortium (BCAC)

We used genotype and clinico-pathological data (database version 12) data from the Breast Cancer Association Consortium (BCAC). The dataset included 1062 breast cancer patients with metastatic primary breast cancer at diagnosis that were genotyped using one of the two different genotyping platforms: iCOGS^12^ and OncoArray^13^, providing genome-wide coverage of common variants. The main analyses were based on imputed variants using the Haplotype Reference Consortium^29^ as reference panel. All patients were women of European ancestry, aged 26–92 years (median: 60) years with metastasized breast cancer at diagnosis. Women were diagnosed between 1979 and 2014, with a median follow-up was three and a half years. Additional details about the genotype data and sample quality control have been described previously^7,27,30^. We only analyzed variants that had a minor allele frequency (MAF) > 0.01 and an imputation quality r^2^ > 0.7 for at least one of the two genotyping platforms (iCOGS or OncoArray). Details about the individual studies included in the analyses, including the array used, associated country and number of patients with metastatic primary breast cancer at diagnosis are given in Supplementary Table 1. The secondary use of data for the study was approved by the Data Access Committee of the BCAC, under the legal provisions of the Memorandum of Understanding and Data Transfer Agreements of Cambridge University which all the contributing institutions, which includes that all contributing institutions provided the data with the appropriate approval of their institutional review boards and informed consent of the participants of the individual studies.

### Statistical and bioinformatic methods

We estimated the association of the germline variants with breast-cancer specific survival using Cox proportional hazards regression. We analyzed separately the OncoArray and iCOGS datasets and combined the estimates using fixed-effect meta-analyses. Follow-up was right censored on the date of death, last date known alive if death did not occur, or at 15 years after diagnosis, whichever came first^27^. Time at risk was calculated from the date of diagnosis with left truncation for prevalent cases. The models were stratified by country and included the first two ancestry informative principal components^12^. We performed the analysis for all breast cancers and for ER-positive and ER-negative tumors separately. To identify evidence of potential cis-regulatory activity, we intersected germline variants with numerous sources of genomic annotation information from primary breast cells (e.g., chromosome conformation, enhancer–promoter correlations, transcription factor and histone modification ChIP-seq). To assess the effect of gene expression on survival we used the Kaplan–Meier plotter on breast tissue data, grade 3 tumors and 15 years of follow-up (180 months)^18^.

### Validation dataset: SNPs to risk of metastasis (StoRM)

To attempt to validate our results we used data from the SNPs to Risk of Metastasis (StoRM) study. StoRM is a multicentric, prospective, cohort study of metastatic breast cancer patients in France that was originally designed to identify genetic and other factors associated with metastatic relapse and survival^19^. Patients aged 18 years or older, with a histologically proven breast cancer that was metastatic for less than 1 year were included. All patients that had another coexisting cancer or another cancer diagnosed within the last 5 years, were excluded from the study. Patients were followed from March 2012 to July 2017. Time to progression on the first metastatic treatment was recorded and patients were followed until death, every 6 months for 3 years, and then annually until July 2017. A total of 293 patients were available for the validation. The median follow-up was of 3.2 years. Because of the short total follow-up time (5 years) and the advanced disease stage of the patients in the cohort, both a recorded progression and/or death were considered as an event in the survival analyses. Of the whole set of 293 patients, 239 had a progression and/or died during the follow-up period.

### Ethical approval

The study was performed in accordance with the Declaration of Helsinki. All individual studies, from which data was used, were approved by the appropriate medical ethical committees and/or institutional review boards. All study participants provided informed consent.

## Supplementary Information

Supplementary Legends.

Supplementary Table 1.

Supplementary Table 2.

Supplementary Table 3.

Supplementary Table 4.
