# Supplementary material for: Germline variants and breast cancer survival in patients with distant metastases at primary breast cancer diagnosis
Source: Sci Rep. 2021 Oct 5;11:19787. doi: 10.1038/s41598-021-99409-3 (PMC8492709; doi:10.1038/s41598-021-99409-3)
Supplement: Supplementary file 1 — Supplementary Legends. [file 41598_2021_99409_MOESM1_ESM.pdf]

Supplementary Information

Germline variants and breast cancer survival in patients with distant metastases at primary breast cancer diagnosis

Maria Escala-Garcia[1], Sander Canisius[1, 2], Renske Keeman[1], Jonathan Beesley[3], Hoda Anton-Culver[4], Volker Arndt[5], Annelie Augustinsson[6], Heiko Becher[7], Matthias W. Beckmann[8], Sabine Behrens[9], Marina Bermisheva[10], Stig E. Bojesen[11-13], Manjeet K. Bolla[14], Hermann Brenner[5, 15, 16], Federico Canzian[17], Jose E. Castelao[18], Jenny Chang-Claude[9, 19], Stephen J. Chanock[20], Fergus J. Couch[21], Kamila Czene[22], Mary B. Daly[23], Joe Dennis[14], Peter Devilee[24, 25], Thilo Dörk[26], Alison M. Dunning[27], Douglas F. Easton[14, 27], Arif B. Ekici[28], A. Heather Eliassen [29, 30], Peter A. Fasching[8, 31], Henrik Flyger[32], Manuela Gago-Dominguez[33, 34], Montserrat García-Closas[20], José A. García-Sáenz[35], Jürgen Geisler[36], Graham G. Giles[37-39], Mervi Grip[40], Melanie Gündert[41-43], Eric Hahnen[44, 45], Christopher A. Haiman[46], Niclas Håkansson[47], Per Hall[22, 48], Ute Hamann[49], Jaana M. Hartikainen[50, 51], Bernadette A.M. Heemskerk-Gerritsen[52], Antoinette Hollestelle[52], Reiner Hoppe[53, 54], John L. Hopper[38], David J. Hunter[30, 55], William Jacot[56], Anna Jakubowska[57, 58], Esther M. John[59, 60], Audrey Y. Jung[9], Rudolf Kaaks[9], Elza Khusnutdinova[10, 61], Linetta B. Koppert[62], Peter Kraft[30, 63], Vessela N. Kristensen[64], Allison W. Kurian[59, 60], Diether Lambrechts[65, 66], Loic Le Marchand[67], Annika Lindblom[68, 69], Robert N. Luben[70, 71], Jan Lubiński[57], Arto Mannermaa[50, 51, 72], Mehdi Manoochehri[49], Sara Margolin[48, 73], Dimitrios Mavroudis[74], Taru A. Muranen[75], Heli Nevanlinna[75], Andrew F. Olshan[76], Håkan Olsson[6], Tjoung-Won Park-Simon[26], Alpa V. Patel[77], Paolo Peterlongo[78], Paul D.P. Pharoah[14, 27], Kevin Punie[79], Paolo Radice[80], Gad Rennert[81], Hedy S. Rennert[81], Atocha Romero[82], Rebecca Roylance[83], Thomas Rüdiger[84], Matthias Ruebner[8], Emmanouil Saloustros[85], Elinor J. Sawyer[86], Rita K. Schmutzler[44, 45, 87], Minouk J. Schoemaker[88], Christopher Scott[89], Melissa C. Southey[37, 39, 90], Harald Surowy[41, 42], Anthony J. Swerdlow[88, 91], Rulla M. Tamimi[30, 92], Lauren R. Teras[77], Emilie Thomas[93], Ian Tomlinson[94, 95], Melissa A. Troester[76], Celine M. Vachon[96], Qin Wang[14], Robert Winqvist[97, 98], Alicja Wolk[47, 99], Argyrios Ziogas[4], kConFab/AOCS Investigators[100], Kyriaki Michailidou[14, 101, 102], Georgia Chenevix-Trench[3], Thomas Bachelot[103] and Marjanka K. Schmidt[1, 104]*.

*Corresponding author

[1] The Netherlands Cancer Institute - Antoni van Leeuwenhoek Hospital, Division of Molecular Pathology, Amsterdam, The Netherlands.

[2] The Netherlands Cancer Institute - Antoni van Leeuwenhoek hospital, Division of Molecular Carcinogenesis, Amsterdam, The Netherlands.

[3] QIMR Berghofer Medical Research Institute, Department of Genetics and Computational Biology, Brisbane, Queensland, Australia.

[4] University of California Irvine, Department of Medicine, Genetic Epidemiology Research Institute, Irvine, CA, USA.

[5] German Cancer Research Center (DKFZ), Division of Clinical Epidemiology and Aging Research, Heidelberg, Germany.
[6] Lund University, Department of Cancer Epidemiology, Clinical Sciences, Lund, Sweden.
[7] University Medical Center Hamburg-Eppendorf, Institute of Medical Biometry and Epidemiology, Hamburg, Germany.
[8] Comprehensive Cancer Center Erlangen-EMN, University Hospital Erlangen, Friedrich-Alexander University Erlangen-Nuremberg (FAU), Department of Gynecology and Obstetrics, Erlangen, Germany.
[9] German Cancer Research Center (DKFZ), Division of Cancer Epidemiology, Heidelberg, Germany.
[10] Ufa Federal Research Centre of the Russian Academy of Sciences, Institute of Biochemistry and Genetics, Ufa, Russia.
[11] Copenhagen University Hospital, Copenhagen General Population Study, Herlev and Gentofte Hospital, Herlev, Denmark.
[12] Copenhagen University Hospital, Department of Clinical Biochemistry, Herlev and Gentofte Hospital, Herlev, Denmark.
[13] University of Copenhagen, Faculty of Health and Medical Sciences, Copenhagen, Denmark.
[14] University of Cambridge, Centre for Cancer Genetic Epidemiology, Department of Public Health and Primary Care, Cambridge, UK.
[15] German Cancer Research Center (DKFZ) and National Center for Tumor Diseases (NCT), Division of Preventive Oncology, Heidelberg, Germany.
[16] German Cancer Research Center (DKFZ), German Cancer Consortium (DKTK), Heidelberg, Germany.
[17] German Cancer Research Center (DKFZ), Genomic Epidemiology Group, Heidelberg, Germany.
[18] Instituto de Investigación Sanitaria Galicia Sur (IISGS), Xerencia de Xestion Integrada de Vigo-SERGAS, Oncology and Genetics Unit, Vigo, Spain.
[19] University Medical Center Hamburg-Eppendorf, Cancer Epidemiology Group, University Cancer Center Hamburg (UCCH), Hamburg, Germany.
[20] National Cancer Institute, National Institutes of Health, Department of Health and Human Services, Division of Cancer Epidemiology and Genetics, Bethesda, MD, USA.
[21] Mayo Clinic, Department of Laboratory Medicine and Pathology, Rochester, MN, USA.
[22] Karolinska Institutet, Department of Medical Epidemiology and Biostatistics, Stockholm, Sweden.
[23] Fox Chase Cancer Center, Department of Clinical Genetics, Philadelphia, PA, USA.
[24] Leiden University Medical Center, Department of Pathology, Leiden, The Netherlands.
[25] Leiden University Medical Center, Department of Human Genetics, Leiden, The Netherlands.
[26] Hannover Medical School, Gynaecology Research Unit, Hannover, Germany.
[27] University of Cambridge, Centre for Cancer Genetic Epidemiology, Department of Oncology, Cambridge, UK.
[28] Comprehensive Cancer Center Erlangen-EMN, University Hospital Erlangen, Friedrich-Alexander University Erlangen-Nuremberg (FAU), Institute of Human Genetics, Erlangen, Germany.
[29] Brigham and Women's Hospital and Harvard Medical School, Channing Division of Network Medicine, Department of Medicine, Boston, MA, USA.
[30] Harvard T.H. Chan School of Public Health, Department of Epidemiology, Boston, MA, USA.
[31] University of California at Los Angeles, David Geffen School of Medicine, Department of Medicine Division of Hematology and Oncology, Los Angeles, CA, USA.
[32] Copenhagen University Hospital, Department of Breast Surgery, Herlev and Gentofte Hospital, Herlev, Denmark.
[33] Instituto de Investigación Sanitaria de Santiago de Compostela (IDIS), Complejo Hospitalario Universitario de Santiago, SERGAS, Fundación Pública Galega de Medicina Xenómica, Santiago de Compostela, Spain.

[34] University of California San Diego, Moores Cancer Center, La Jolla, CA, USA.
[35] Instituto de Investigación Sanitaria San Carlos (IdISSC), Centro Investigación Biomédica en Red de Cáncer (CIBERONC), Medical Oncology Department, Hospital Clínico San Carlos, Madrid, Spain.
[36] Akershus University Hospital, Department of Oncology, Lørenskog, Norway.
[37] Cancer Council Victoria, Cancer Epidemiology Division, Melbourne, Victoria, Australia.
[38] The University of Melbourne, Centre for Epidemiology and Biostatistics, Melbourne School of Population and Global Health, Melbourne, Victoria, Australia.
[39] Monash University, Precision Medicine, School of Clinical Sciences at Monash Health, Clayton, Victoria, Australia.
[40] University of Oulu, Department of Surgery, Oulu University Hospital, Oulu, Finland.
[41] German Cancer Research Center (DKFZ), Molecular Epidemiology Group, C080, Heidelberg, Germany.
[42] University of Heidelberg, Molecular Biology of Breast Cancer, University Womens Clinic Heidelberg, Heidelberg, Germany.
[43] Helmholtz Zentrum München, German Research Center for Environmental Health, Institute of Diabetes Research, Neuherberg, Germany.
[44] Faculty of Medicine and University Hospital Cologne, University of Cologne, Center for Familial Breast and Ovarian Cancer, Cologne, Germany.
[45] Faculty of Medicine and University Hospital Cologne, University of Cologne, Center for Integrated Oncology (CIO), Cologne, Germany.
[46] University of Southern California, Department of Preventive Medicine, Keck School of Medicine, Los Angeles, CA, USA.
[47] Karolinska Institutet, Institute of Environmental Medicine, Stockholm, Sweden.
[48] Sšdersjukhuset, Department of Oncology, Stockholm, Sweden.
[49] German Cancer Research Center (DKFZ), Molecular Genetics of Breast Cancer, Heidelberg, Germany.
[50] University of Eastern Finland, Translational Cancer Research Area, Kuopio, Finland.
[51] University of Eastern Finland, Institute of Clinical Medicine, Pathology and Forensic Medicine, Kuopio, Finland.
[52] Erasmus MC Cancer Institute, Department of Medical Oncology, Rotterdam, The Netherlands.
[53] Dr. Margarete Fischer-Bosch-Institute of Clinical Pharmacology, Stuttgart, Germany.
[54] University of Tübingen, Tübingen, Germany.
[55] University of Oxford, Nuffield Department of Population Health, Oxford, UK.
[56] Institut du Cancer de Montpellier, Montpellier University, Montpellier, France.
[57] Pomeranian Medical University, Department of Genetics and Pathology, Szczecin, Poland.
[58] Pomeranian Medical University, Independent Laboratory of Molecular Biology and Genetic Diagnostics, Szczecin, Poland.
[59] Stanford Cancer Institute, Stanford University School of Medicine, Department of Medicine, Division of Oncology, Stanford, CA, USA.
[60] Stanford University School of Medicine, Department of Epidemiology & Population Health, Stanford, CA, USA.
[61] Bashkir State University, Department of Genetics and Fundamental Medicine, Ufa, Russia.
[62] Erasmus MC Cancer Institute, Department of Surgical Oncology, Family Cancer Clinic, Rotterdam, The Netherlands.
[63] Harvard T.H. Chan School of Public Health, Program in Genetic Epidemiology and Statistical Genetics, Boston, MA, USA.
[64] Oslo University Hospital and University of Oslo, Department of Medical Genetics, Oslo, Norway.
[65] VIB Center for Cancer Biology, Leuven, Belgium.

[66] University of Leuven, Laboratory for Translational Genetics, Department of Human Genetics, Leuven, Belgium.
[67] University of Hawaii Cancer Center, Epidemiology Program, Honolulu, HI, USA.
[68] Karolinska Institutet, Department of Molecular Medicine and Surgery, Stockholm, Sweden.
[69] Karolinska University Hospital, Department of Clinical Genetics, Stockholm, Sweden.
[70] NIHR Biomedical Research Centre, Moorfields Eye Hospital NHS Foundation Trust and UCL Institute of Ophthalmology, London, England, UK.
[71] Medical Research Council (MRC) Epidemiology Unit, University of Cambridge, Cambridge, United Kingdom.
[72] Kuopio University Hospital, Biobank of Eastern Finland, Kuopio, Finland.
[73] Karolinska Institutet, Department of Clinical Science and Education, Sšdersjukhuset, Stockholm, Sweden.
[74] University Hospital of Heraklion, Department of Medical Oncology, Heraklion, Greece.
[75] University of Helsinki, Department of Obstetrics and Gynecology, Helsinki University Hospital, Helsinki, Finland.
[76] University of North Carolina at Chapel Hill, Department of Epidemiology, Gillings School of Global Public Health and UNC Lineberger Comprehensive Cancer Center, Chapel Hill, NC, USA.
[77] American Cancer Society, Department of Population Science, Atlanta, GA, USA.
[78] IFOM - the FIRC Institute of Molecular Oncology, Genome Diagnostics Program, Milan, Italy.
[79] Leuven Cancer Institute, University Hospitals Leuven, Department of General Medical Oncology and Multidisciplinary Breast Centre, Leuven, Belgium.
[80] Fondazione IRCCS Istituto Nazionale dei Tumori (INT), Unit of Molecular Bases of Genetic Risk and Genetic Testing, Department of Research, Milan, Italy.
[81] Carmel Medical Center and Technion Faculty of Medicine, Clalit National Cancer Control Center, Haifa, Israel.
[82] Hospital Universitario Puerta de Hierro, Medical Oncology Department, Madrid, Spain.
[83] UCLH Foundation Trust, Department of Oncology, London, UK.
[84] Staedtisches Klinikum Karlsruhe, Institute of Pathology, Karlsruhe, Germany.
[85] University Hospital of Larissa, Department of Oncology, Larissa, Greece.
[86] King's College London, School of Cancer & Pharmaceutical Sciences, Comprehensive Cancer Centre, Guy's Campus, London, UK.
[87] Faculty of Medicine and University Hospital Cologne, University of Cologne, Center for Molecular Medicine Cologne (CMMC), Cologne, Germany.
[88] The Institute of Cancer Research, Division of Genetics and Epidemiology, London, UK.
[89] Mayo Clinic, Department of Health Sciences Research, Rochester, MN, USA.
[90] The University of Melbourne, Department of Clinical Pathology, Melbourne, Victoria, Australia.
[91] The Institute of Cancer Research, Division of Breast Cancer Research, London, UK.
[92] Weill Cornell Medicine, Department of Population Health Sciences, New York, NY, USA.
[93] Plateforme de Bioinformatique Gilles Thomas, Centre de recherche en cancérologie de Lyon, Fondation Synergie Lyon Cancer, Université Claude Bernard Lyon 1, Lyon, France
[94] University of Birmingham, Institute of Cancer and Genomic Sciences, Birmingham, UK.
[95] University of Oxford, Wellcome Trust Centre for Human Genetics and Oxford NIHR Biomedical Research Centre, Oxford, UK.
[96] Mayo Clinic, Department of Health Science Research, Division of Epidemiology, Rochester, MN, USA.
[97] University of Oulu, Laboratory of Cancer Genetics and Tumor Biology, Cancer and Translational Medicine Research Unit, Biocenter Oulu, Oulu, Finland.

[98] Northern Finland Laboratory Centre Oulu, Laboratory of Cancer Genetics and Tumor Biology, Oulu, Finland.
[99] Uppsala University, Department of Surgical Sciences, Uppsala, Sweden.
[100] Peter MacCallum Cancer Center, Melbourne, Victoria, Australia.
[101] The Cyprus Institute of Neurology & Genetics, Biostatistics Unit, Nicosia, Cyprus.
[102] The Cyprus Institute of Neurology & Genetics, Cyprus School of Molecular Medicine, Nicosia, Cyprus.
[103] Centre Léon Bérard, Département de Cancérologie Médicale, Lyon, France
[104] The Netherlands Cancer Institute - Antoni van Leeuwenhoek hospital, Division of Psychosocial Research and Epidemiology, Amsterdam, The Netherlands.

Supplementary Tables

Supplementary Table 1. Data description and design of the studies included in the analysis.

Supplementary Table 2. Results of the top 100 variants in the analysis of all breast cancer-specific survival for patients with metastasis at primary breast cancer diagnosis.

Supplementary Table 3. Results of the top 100 variants in the analysis of ER-positive breast cancer-specific survival for patients with metastasis at primary breast cancer diagnosis.

Supplementary Table 4. Results of the top 100 variants in the analysis of ER-negative breast cancer-specific survival for patients with metastasis at primary breast cancer diagnosis.
